# A Granulin-Like Growth Factor Secreted by the Carcinogenic Liver Fluke, *Opisthorchis viverrini*, Promotes Proliferation of Host Cells

**DOI:** 10.1371/journal.ppat.1000611

**Published:** 2009-10-09

**Authors:** Michael J. Smout, Thewarach Laha, Jason Mulvenna, Banchob Sripa, Sutas Suttiprapa, Alun Jones, Paul J. Brindley, Alex Loukas

**Affiliations:** 1 Division of Infectious Diseases, Queensland Institute of Medical Research, Queensland, Australia, and School of Population Health, The University of Queensland, Queensland, Australia; 2 Department of Parasitology, Khon Kaen University, Khon Kaen, Thailand; 3 Department of Pathology, Khon Kaen University, Khon Kaen, Thailand; 4 Institute for Molecular Biosciences, The University of Queensland, Queensland, Australia; 5 Department of Microbiology, Immunology and Tropical Medicine, George Washington University, Washington, D. C., United States of America; University of Pennsylvania, United States of America

## Abstract

The human liver fluke, *Opisthorchis viverrini*, infects millions of people throughout south-east Asia and is a major cause of cholangiocarcinoma, or cancer of the bile ducts. The mechanisms by which chronic infection with *O. viverrini* results in cholangiocarcinogenesis are multi-factorial, but one such mechanism is the secretion of parasite proteins with mitogenic properties into the bile ducts, driving cell proliferation and creating a tumorigenic environment. Using a proteomic approach, we identified a homologue of human granulin, a potent growth factor involved in cell proliferation and wound healing, in the excretory/secretory (ES) products of the parasite. *O. viverrini* granulin, termed *Ov*-GRN-1, was expressed in most parasite tissues, particularly the gut and tegument. Furthermore, *Ov*-GRN-1 was detected *in situ* on the surface of biliary epithelial cells of hamsters experimentally infected with *O. viverrini*. Recombinant *Ov*-GRN-1 was expressed in *E. coli* and refolded from inclusion bodies. Refolded protein stimulated proliferation of murine fibroblasts at nanomolar concentrations, and proliferation was inhibited by the MAPK kinase inhibitor, U0126. Antibodies raised to recombinant *Ov*-GRN-1 inhibited the ability of *O. viverrini* ES products to induce proliferation of murine fibroblasts and a human cholangiocarcinoma cell line *in vitro*, indicating that *Ov*-GRN-1 is the major growth factor present in *O. viverrini* ES products. This is the first report of a secreted growth factor from a parasitic worm that induces proliferation of host cells, and supports a role for this fluke protein in establishment of a tumorigenic environment that may ultimately manifest as cholangiocarcinoma.

## Author Summary

The oriental liver fluke is endemic through South-East Asia and is the major cause of cause of liver cancer in north-eastern Thailand. The molecules that are secreted by the parasite cause cells to multiply quicker than they normally would, and excessive cell growth is a key stage in the initiation of many cancers. We identified a secreted protein from the fluke, termed granulin, which has a similar structure to a human growth factor associated with many aggressive cancers. Granulin is secreted by the parasite into the bile ducts where it causes host cells to proliferate. The proliferative activity of fluke secreted proteins was blocked by antibodies against granulin, indicating that it is the major cell growth-inducing molecule released by the parasite. Identifying the function of granulin will enable us to understand how and why this debilitating yet neglected pathogen causes cancer in so many people in South-East Asia. This and future work will contribute towards the development of new strategies to reduce both parasite prevalence and the incidence of the most fatal of liver cancers in Thailand.

## Introduction

Cholangiocarcinoma (CCA), or cancer of the bile ducts, is prevalent in people from Thailand and Laos whose staple diet includes uncooked fish which harbour the liver fluke, *Opisthorchis viverrini*, the main risk factor for this cancer in the region [1]. There is no stronger link between a parasite and cancer than that between *O. viverrini* and CCA - indeed WHO data suggest that as many as one-third of the nine million infected people will contract cancer [2]. This is a striking figure compared to data from other carcinogenic microbes, such as *Helicobacter pylori*, human papilloma virus and the hepatitis viruses, where less than one percent of infected individuals develop infection-related cancers [2],[3].

For opisthorchiasis, *in vivo* studies in hamsters and *in vitro* investigations have indicated that the fluke's excretory/secretory (ES) products, metabolic products excreted and secreted into the external environment from the excretory openings and epithelial surface (tegument), include mitogens that likely play a role in the initiation of CCA in infected humans and experimentally infected hamsters [4],[5]. To gain a better understanding of the host-parasite interactions underlying the molecular pathogenesis of opisthorchiasis, we screened both the transcriptome [6] and the ES proteome (J. Mulvenna et al., unpublished) of the fluke for genes encoding proteins with ontologies that were associated with human cancers. A homologue of human granulin, a secreted growth factor implicated in many aggressive and invasive cancers, was identified.

The granulin domain consists of 12 highly conserved cysteines and is found in diverse phyla from eubacteria to humans, and subsequently has many synonyms [7]. Compounding the confusion, the term granulin can also refer to the small 6–10 kDa granulin domain (also named epithelins or GEM∶granulin/epithelin modules) found in the majority of animals, or the vertebrate protein, progranulin (PGRN), which in mammals is a large 60–90 kDa glycoprotein containing seven tandemly repeated granulin motifs [8]. PGRN protein is also known as PC cell-derived growth factor (PCDGF), proepithelin (PEPI), Granulin/epithelin precursor (GEP), GP88, acrogranin, granulin or epithelin precursor [9]. Herein we will refer to the large multihomodomain form from vertebrates as PGRN, and granulin (GRN) will refer to the individual granulin domains.

There is a broad distribution of PGRN in human organs and tissues, and elevated levels of mRNA are found in organs with neuronal cells (cerebellum), hematopoietic stem cells (spleen) and rapidly dividing epithelium (skin, gastrointestinal tract and wounded epithelia) [10],[11]. Numerous functions for GRNs have been reported but the roles in cell cycle control and wound healing are noteworthy [8]. Numerous mutations have been observed within the human PGRN gene with many linked to psychiatric disorders including Alzheimer's disease and frontotemporal dementia [12],[13].

Over-expression of PGRN is linked to tumorigenesis in numerous human tissues, including liver cancers, and is associated with an aggressive and invasive tumour phenotype [14],[15]. GRN is a potent proliferative agent but has other pro-tumor qualities that are not yet well characterized. It may promote carcinoma progression by promoting angiogenesis, insensitivity to apoptosis, promotion of tumor invasion and anchorage independence which all support tumor expansion in the unfavorable interstitial environment [7],[16],[17]. Preventing over-expression of PGRN in a range of tumor types, either through gene silencing or neutralizing antibodies, reduces or entirely inhibits tumor progression [18]. Over-expression of PGRN is an indicator of poor prognosis for a range of cancer types, and anti-GRN antibodies have been successfully employed in mice as therapy for hepatocellularcarcinoma (HCC) [19].

Large-scale gene sequencing efforts have revealed GRN homologues in the majority of parasitic phyla [20],[21],[22]. Like free-living eukaryotes, parasitic helminths probably utilize GRN to regulate growth and development of their own cells. By contrast, here we describe the detection of GRN in the ES products of *O. viverrini* and its binding to mammalian biliary epithelial cells *in situ*. Furthermore, recombinant *O. viverrini* GRN stimulated proliferation of fibroblasts, whereas antibodies against the recombinant GRN inhibited the ability of ES products to promote proliferation. Together these findings support a role for this fluke protein in establishment of a tumorigenic environment that may ultimately manifest as CCA.

## Results

### *Ov*-GRN-1 is a homologue of a human growth factor associated with cancer progression

**Figure 1:**
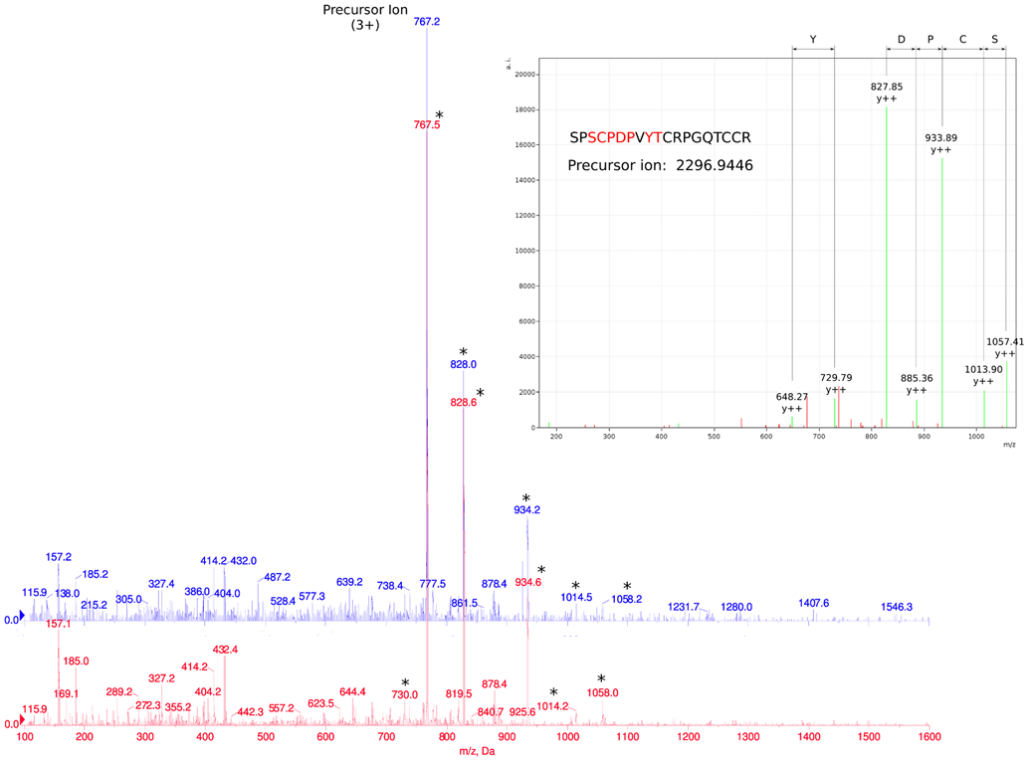
Comparison of multiple-reaction monitoring (MRM) MS/MS fragmentation spectra targeted against a 19 amino acid *Ov*-GRN-1 peptide identified during the course of shotgun proteomics. Recombinant *Ov*-GRN-1 (red) and *O. viverrini* ES products (blue) were analyzed in separate MRM experiments targeted against a previously identified *Ov*-GRN-1 peptide. In both experiments the target precursor ion eluted from the reverse-phase column at the same time and produced identical MS/MS fragmentation patterns. The MS/MS spectrum from the original identification is shown in the inset and the identified fragments are labeled. Peaks corresponding to the MS/MS spectra are highlighted with an asterisk on the MRM fragmentation spectrum along with the triple charged precursor ions.

Characterization of the protein profile of *O. viverrini* adult worm ES products using LC-MS/MS revealed a 19 amino acid peptide, with a MOWSE score of 50 (delta error -0.0464) (Figure 1 inset), that matched to a single contig encoding a protein with sequence similarity to human granulin (not shown). The cDNA was termed *Ov-grn-1* and its protein product *Ov*-GRN-1; the sequences were submitted to GenBank under accession number FJ436341. Verification of the MS/MS identification was then performed using multiple-reaction monitoring (MRM) transitions targeted against the peptide identified in the shotgun proteomics. Firstly, a tryptic digest of *O. viverrini* ES products, collected after one day of culture, was analyzed and the target peptide identified and fragmented. Next, recombinant *Ov*-GRN-1 in water was digested and analyzed in the same fashion. The target peptide from the ES sample was identified at the same elution time and with an identical product ion spectrum as that generated for recombinant *Ov*-GRN-1 (Figure 1). The peptide observed showed very abundant y-ion fragments, as expected, on the C-terminal side of the peptide. The peptide also revealed a missed trypsin cleavage at the Arg residue, due to the Pro residue on its C-terminal side. The combination of the initial MS/MS data, elution time of the target peptide in both samples and the similarity of MRM fragmentation patterns strongly supports the presence of *Ov*-GRN-1 in the ES products of *O. viverrini*.

**Figure 2:**
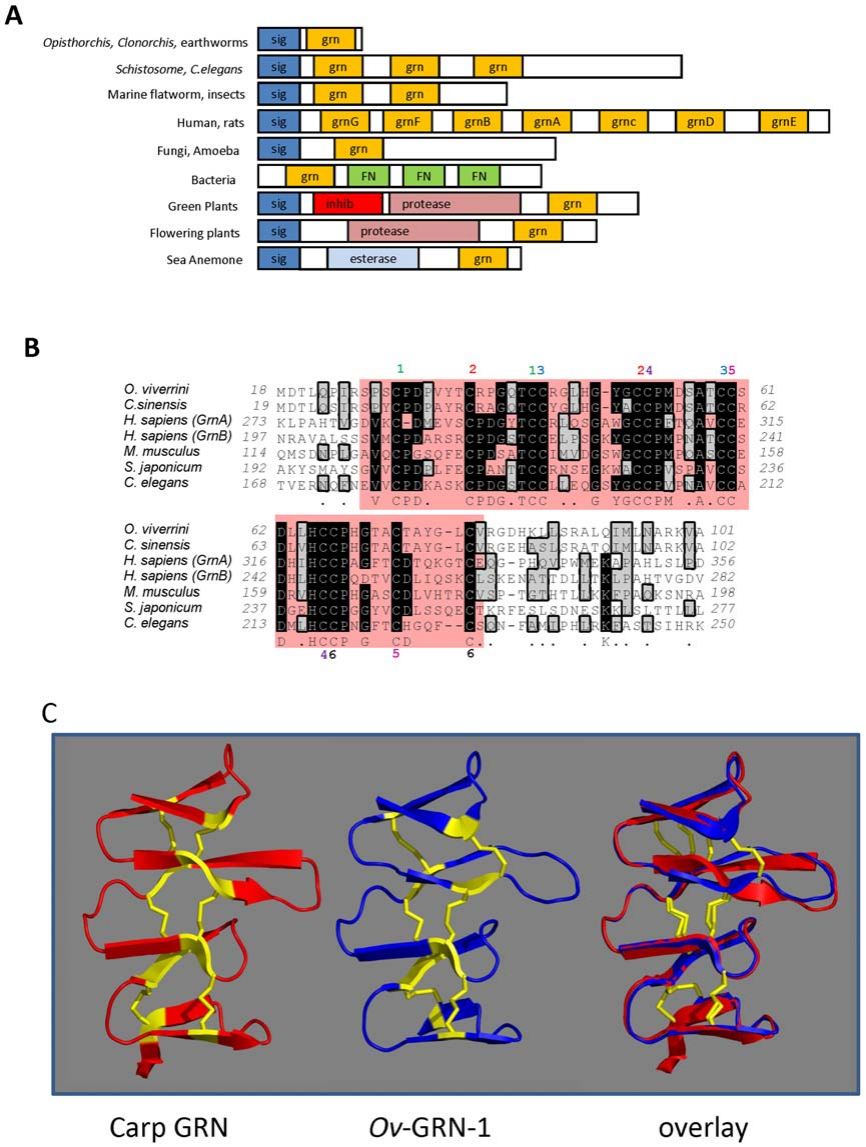
*Ov*-GRN-1 is a secreted growth factor belonging to the granulin family. Panel A - structural architecture of proteins containing granulin domains. Grn or GrnA-G: granulin domains, Inhib: cathepsin propeptide inhibitor, FN: Fibronectin type III, Sig: Secretion Signal sequence, Protease: Papain family cysteine protease, Esterase: SGNH hydrolase type esterase. Sequence accession numbers are as described in the legend for Figure 2, with structural architecture obtained from Interpro 18.0 database. Panel B - multiple sequence alignment of *Ov*-GRN-1 and granulin domains from other organisms. Identical amino acids are in black boxes and similar amino acids are in gray boxes. The granulin core domain is highlighted by a pink box. Theoretical disulphide bonds are numbered one to six above each cysteine residue. GenBank accession numbers are as follows: *Opisthorchis viverrini Ov*-GRN-1- FJ436341*; *Clonorchis sinensis* (parasitic liver fluke) - AT006891*; *Schistosoma japonicum* (parasitic blood fluke) - AY810079*; *Caenorhabditis elegans* (free living roundworm) - NP_492982*; *Homo sapiens* (human) granulin domains A and B - AW51601; *Mus musculus* (mouse) - NM_008175*. *Amino acid numbering is from the initiator (or predicted initiator) methionine. Panel C - molecular model of the granulin core domain of *Ov*-GRN-1 based on the nuclear magnetic resonance structure of carp granulin [23] and an overlay where *Ov*-GRN-1 is superimposed on carp granulin. Disulphide bonds are highlighted in yellow.

*Ov*-GRN-1, like homologues from the related liver fluke *Clonorchis sinensis* and earthworms, has an N-terminal signal peptide followed by a single GRN core domain. Most other proteins containing a GRN domain consist of multiple GRN domains (PGRN) or at least one GRN domain fused to other domains including proteases, protease inhibitors and fibronectin (Figure 2A). *Ov*-GRN-1 consists of a predicted secretion signal peptide followed by an 84 amino acid GRN domain of 9.04 kDa with twelve conserved cysteines. Whereas no *N*-linked glycosylation sites were predicted, four putative *O*-glycosylation sites were identified at Ser-26, Thr-35, Thr-41 and Ser-61. The core GRN domain of *Ov*-GRN-1 shared 43.6% identity at the amino acid level with granulin F, the closest human homologue, and 85% identity with an EST from the related liver fluke, *Clonorchis sinensis* (Figure 2B). Data from the few GRN structures available suggest that *Ov*-GRN-1 adopts the general GRN fold and disulphide bonding pattern akin to carp GRN, the only complete GRN structure available to date [23]. The NMR derived structure of carp (*Cyprinus carpio*) granulin [23] was used as the template on which to build a molecular model of *Ov*-GRN-1 (Figure 2C). The two proteins shared 32% identity over their granulin core domains. The lowest energy structure from 50 calculated using MODELLER was selected as an approximation of the structure of *Ov*-GRN-1. The model contained no violations of distance restraints and the Ramachandran plot, calculated with PRO-CHECK-NMR [24], showed a single residue in the disallowed regions.

### Phylogenetic relationships

**Figure 3:**
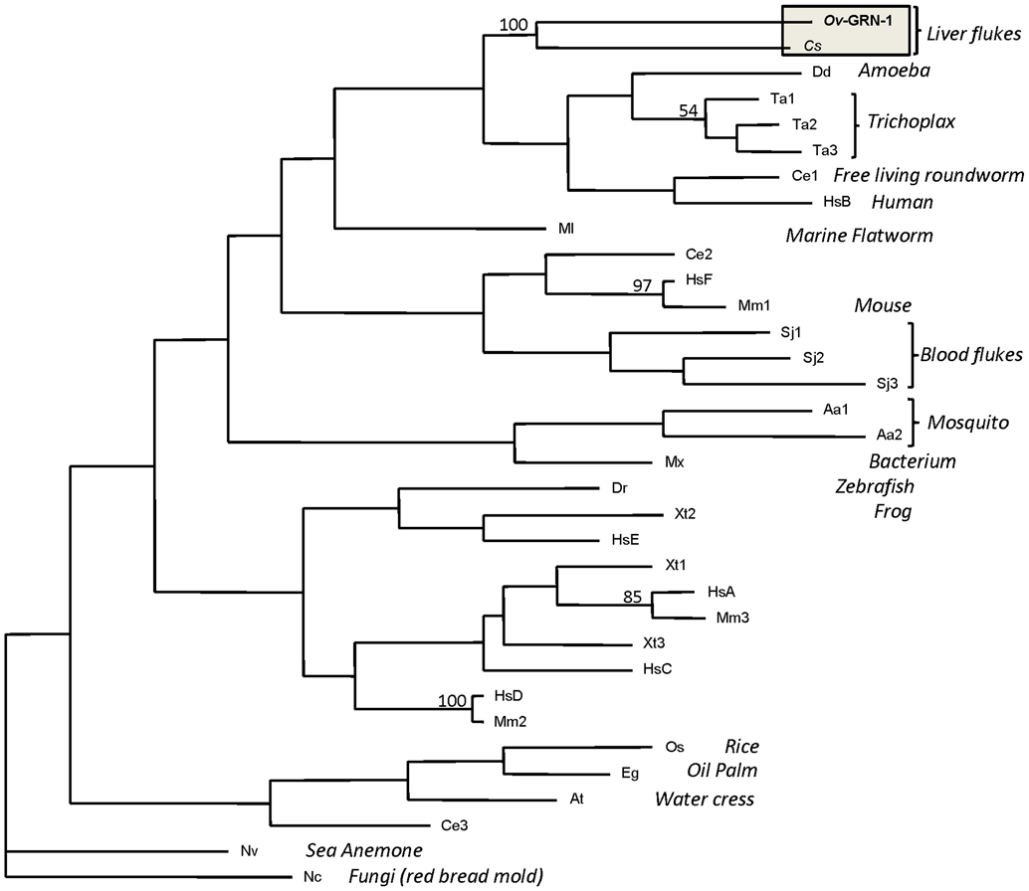
Neighbor joining phylogenetic tree for the granulin core domains from a range of phyla. Numbers on the branches are bootstrap values that obtained greater than 50% support from 1000 replicates. GenBank accession numbers are as follows: *Ov*-GRN-1 - FJ436341; Cs- *Clonorchis sinensis* (parasitic liver fluke) AT006891, Sj - *Schistosoma japonicum* (parasitic blood fluke) AAX25968; Mx - *Myxococcus xanthus* (bacterium) Q1CVL1; Nc - *Neurospora crassa* (fungus) Q7S2H4; Dr - *Danio rerio* (zebrafish) A8E5C4; Xt - *Xenopus tropicalis* (frogs) CAJ82256; Nv - *Nematostella vectensis* (sea anemone) A7SM19; Aa - *Aedes albopictus* (mosquito) B0FU00; Os - *Oryza sativa* (rice) Q7XR52; Eg - *Elaeis guineensis* var. tenera (oil palm) A6N8F8; Ta - *Trichoplax adhaerens* (tablet animals) B3RQV7; Dd - *Dictyostelium discoideum* (amoeba) Q54QR7; At - *Arabidopsis thaliana* (mouse-ear cress) Q9LT78; Ce - *Caenorhabditis elegans* (free living roundworm) BAE35565; Hs - *Homo sapiens* (human) EAW51601; Mm - *Mus musculus* (mouse) NP_492982.

*Ov*-GRN-1 grouped very closely with its orthologue from the related liver fluke, *C. sinensis*, a parasite that has also been implicated as a cause of human CCA [25], and this clade obtained 100% bootstrap support (Figure 3). GRNs from the placozoan *Trichoplax*, slime mould, the free-living nematode *Caenorhabditis elegans* and human granulin B also formed a clade with the liver fluke GRNs, although this did not obtain bootstrap support of greater than 50%. Interestingly, the blood fluke (*Schistosoma*) GRNs did not group closely with *Ov*-GRN-1 – most *O. viverrini* genes share greater sequence identity with other platyhelminth genes [6] than they do with genes from other phyla, suggesting that the phylogeny presented here reflects functional protein relationships rather than taxonomic relationships. It is also noteworthy that the schistosome GRN domains were probably derived from a multi-domain PGRN.

### *Ov-grn-1* is expressed throughout development of the liver fluke

Reverse transcription PCR using RNA from different *Opisthorchis* life cycle stages amplified a product of the expected size, ∼300 bp, in all developmental stages tested (Figure S1), indicating that *Ov-grn-1* was constitutively expressed throughout the developmental cycle of the liver fluke. An amplicon was not detected in the absence of reverse transcriptase enzyme (not shown), confirming the absence of contaminating genomic DNA. The constitutively expressed actin gene served as a control and was expressed in all stages.

### Expression of recombinant *Ov*-GRN-1

**Figure 4:**
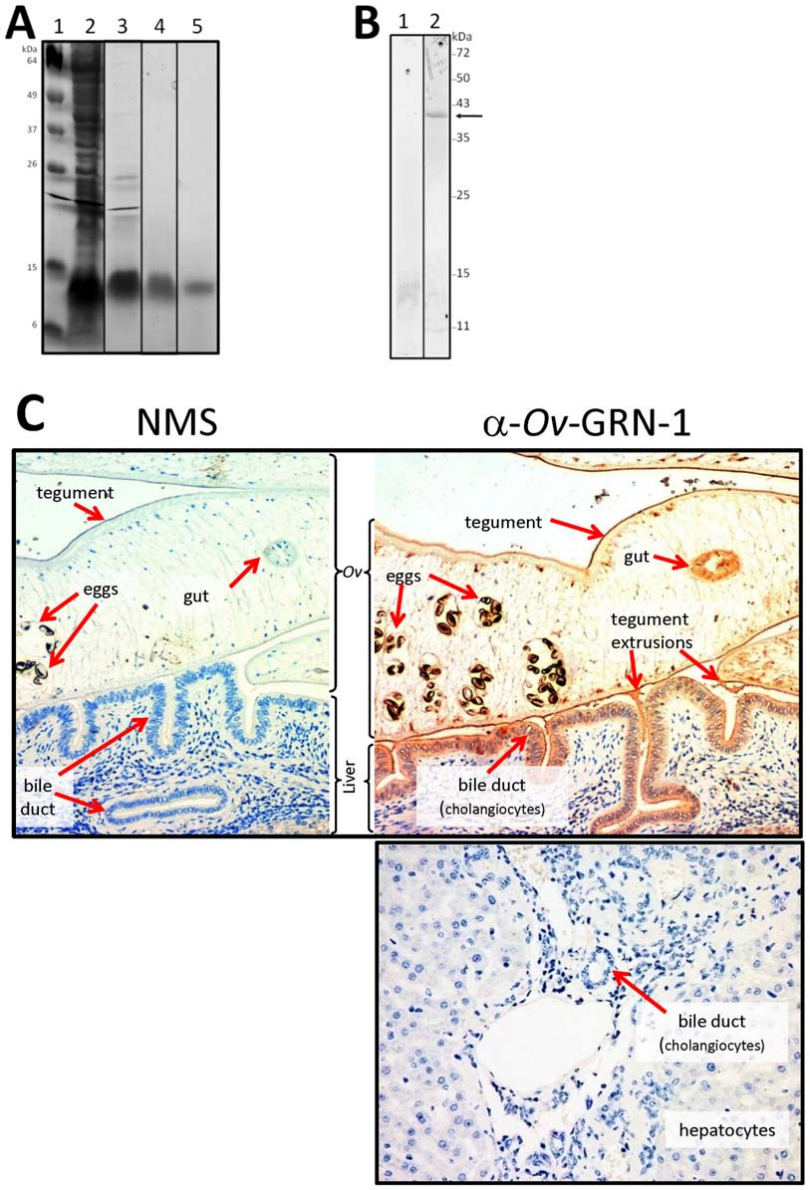
Recombinant *Ov*-GRN-1e expressed in *E. coli* and verification of anti-*Ov*-GRN-1 antibodies. Panel A: 15% SDS PAGE gel stained with Imperial Blue showing purification of *Ov*-GRN-1e. Lane 1: Benchmark Marker (Invitrogen), 2: French pressed total cell pellet, 3: Insoluble fraction, 4: column eluate containing purified recombinant *Ov*-GRN-1e, 5: refolded *Ov*-GRN-1e. Panel B: Recognition of native *Ov*-GRN-1 in *O. viverrini* somatic protein extract by mouse anti-*Ov*-GRN-1s IgG. SDS-PAGE was conducted under native conditions and the blot was probed with control IgG (1) or anti-*Ov*-GRN-1s IgG (2). The arrow highlights the major band at 38 kDa. Molecular masses (kDa) are indicated on the right. Panel C: Immunolocalization of *Ov*-GRN-1 in histological sections of adult *O. viverrini* in the bile ducts of experimentally infected hamsters. The left panel was probed with control IgG; the right panel was probed with anti-*Ov*-GRN-1e IgG. The bottom panel shows a liver section from an uninfected hamster that was probed with anti-*Ov*-GRN-1e IgG. Peroxidase staining is revealed as a brown/rust colored deposit in panel B and Mayer's Haematoxylin counterstained the nuclei in blue. Red arrows highlight the regions within in the *O. viverrini* parasite and bile duct tissue that stained positive for *Ov*-GRN-1.

*Ov*-GRN-1 was expressed in *E. coli* and in *Sf*9 insect cells. We herein refer to the *E. coli*-derived recombinant protein as *Ov*-GRN-1e and *Sf*9-derived protein as *Ov*-GRN-1s. Soluble 6×His tagged *Ov*-GRN-1s protein with a molecular mass of ∼14 kDa was expressed in *Sf*9 cells at a yield of ≤200 µg purified protein per litre of culture medium. A combination of cation exchange and Ni-NTA affinity chromatographies followed by a second Ni-NTA purification resulted in recombinant granulin that appeared to be greater than 95% pure based on SDS-PAGE gels (not shown). *Ov*-GRN-1e was highly expressed (up to 60 mg/L) in each of three *E. coli* cell lines (BL21, Rosetta, Rosetta-gami) but in each case the *Ov*-GRN-1e was insoluble and required urea (or other chaotropic agents) to solubilise. Different induction times and temperatures were assessed, and none of these variables promoted the solubility of the recombinant *Ov*-GRN-1e. To obtain denatured recombinant protein, BL21 *E. coli* cells transformed with pET41a encoding the *Ov*-GRN-1 ORF were grown at 37°C and induced for 16 h. Recombinant protein was purified on Ni-NTA resin under denaturing conditions to yield >95% pure protein (Figure 4A). Refolding of the purified denatured protein was undertaken, and the best conditions identified yielded ∼15% recovery of soluble protein by refolding in 20 mM Tris pH 7.5, 1 mM CaCl_2_ for 24 hr.

### Antibodies against *Ov*-GRN-1 recognize native epitopes

IgG antibodies raised against recombinant *Ov*-GRN-1e and GRN-1s proteins in mice were employed to probe the recombinant immunogens and *Opisthorchis* somatic adult extract (SAE) by Western blotting under both native and denaturing/reducing conditions. Bands were only visible when SAE was probed under native conditions with both anti-GRN-1s and anti-GRN-1e IgGs, with a strong band visible at 38 kDa, higher than the predicted 9 kDa mass of the monomeric protein (Figure 4B). Neither antibody bound to any proteins under reducing/denaturing conditions (not shown), suggesting that conformational epitopes were the target of anti-GRN-1 antibodies, and that the native protein might form homo-multimers or form complexes with other proteins under native conditions. Control antibodies did not produce any bands under native or denaturing/reducing conditions. Anti-GRN-1s IgG was used to localize the sites of expression within adult *O. viverrini* and in the surrounding bile ducts of an experimentally infected hamster. *Ov*-GRN-1 exhibited ubiquitous expression through all tissues, particularly in the gut, tegument and tegument extrusions of the adult worm (Figure 4C, right upper panel). Interestingly, the protein was also strongly detected in the bile duct epithelial cells in close proximity to the liver fluke. Control mouse IgG did not bind to any tissue or structures in the fluke or hamster tissues (Figure 4C, left panel). Additionally anti-*Ov*-GRN-1s IgG showed no affinity for host granulin as indicated by a lack of staining in uninfected hamster liver (Figure 4C, lower right panel)

### *O. viverrini* ES products and recombinant *Ov*-GRN-1e induce cell proliferation

**Figure 5:**
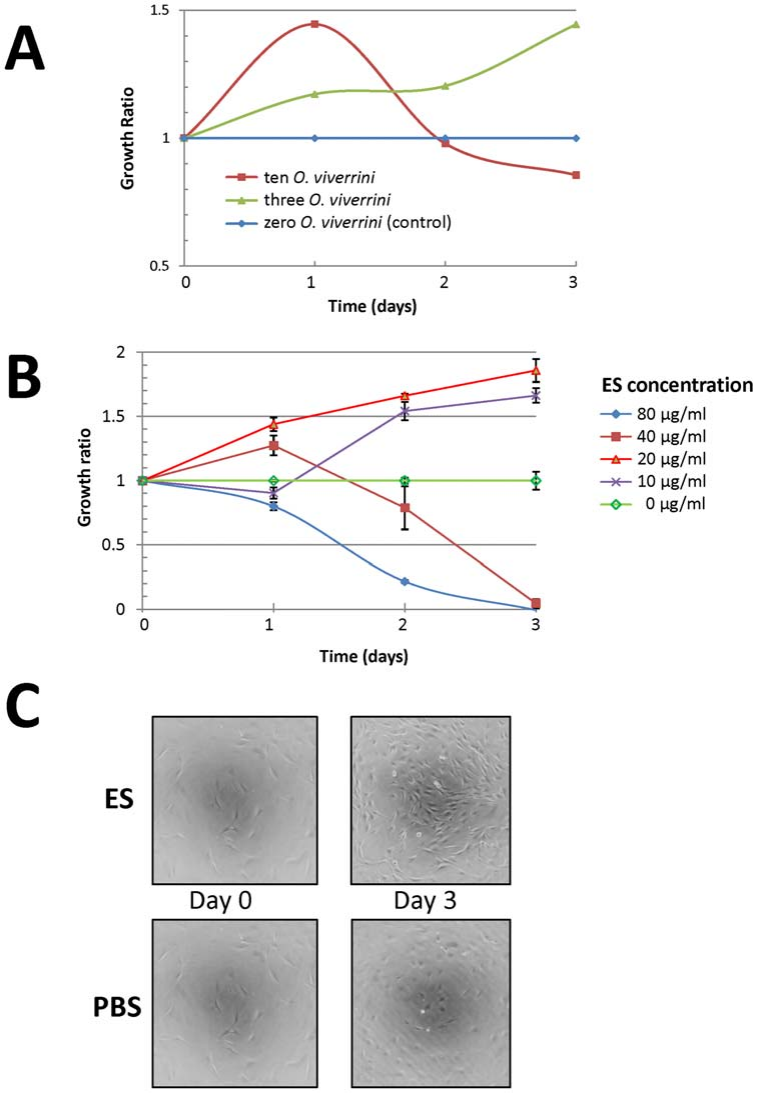
Proliferation of host cells in response to *O. viverrini* proteins. Panel A: Non-contact co-culture of 3 or 10 living adult *O. viverrini* worms with KKU100 human biliary cancer cell line over time. Growth is expressed as a ratio of cells cultured in the presence or absence of *O. viverrini*. Cell numbers were manually counted using a hemocytometer. Panel B: Effect of different concentrations of *O. viverrini* adult worm ES on the growth of NIH-3T3 mouse fibroblasts over time. Cell growth was measured using WST-1 metabolic assay as a marker of mitochondrial enzyme activity. Growth is expressed as a ratio compared to cells that did not receive ES with error bars indicating one standard deviation. Panel C: Morphology changes of NIH-3T3 mouse fibroblasts cultured in the presence or absence of *O. viverrini* adult worm ES (20 µg/ml) after 3 days.

Others have shown that *O. viverrini* causes proliferation of fibroblasts and the KKU-100 CCA cell line when these cells are co-cultured in the presence of live adult *O. viverrini* in a non-contact format [4],[26]. We reproduced these findings (Figures 5A and S2A) and proceeded to show that soluble ES products from *O. viverrini* stimulated proliferation of NIH-3T3 fibroblasts (Figures 5B, S2B and S3) and the KKU-100 CCA line (not shown). Cell proliferation was measured using two distinct approaches. WST-1 is a measure of metabolic activity of cells, and although it is routinely used to measure cell growth over time, metabolic variations in cells exposed to different conditions (e.g. in the presence of ES products) can mask changes in real cell numbers. We also assessed the growth of cells using a real time index of measurement to corroborate the proliferation quantified by the WST-1 assay using an xCELLigence system. The cell index readout is a real time measure of conductivity which is indicative of cell surface area in contact with the gold electrodes covering the plate surface [27]. We optimised the conditions for ES-induced cell growth to permit a thorough assessment of the effects of ES products (and other treatments) over time. Cells were seeded at an adequate density to determine growth over 3 days in a reliable manner before reaching confluence. Conditions that resulted in a minimum of two-fold growth of NIH-3T3 fibroblasts between samples treated with and without ES over 3 days were determined in the presence of increasing concentrations of bovine calf serum (BCS) in a 96 well plate – final conditions were 0% BCS seeded at 6000 cells/well, 2% BCS at 2000 cells/well, 5% BCS at 700 cells/well and 10% BCS at 300 cells/well. The optimal conditions identified for detection of approximately two-fold proliferation of NIH-3T3 fibroblasts induced by addition of ES compared with an equal volume of PBS (control) were as follows: 2,000 cells seeded per well and cultured for 3–8 h in DMEM containing antibiotic/antimycotic at 37°C in 95% air/5% CO_2_ and 2% BCS prior to addition of ES products (20 µl) to a final concentration of 20 µg/ml. Cells were cultured in the presence of ES or PBS and cell numbers were determined using the WST-1 dye procedure. Addition of ES to cells grown in all serum concentrations tested resulted in changes in cell growth and morphology; Figure 5C presents the results of cell growth in the presence of 2% BCS. The flattened fibroblastic shape of 3T3 cells changed upon addition of ES products, resulting in a longer, narrower and more refractive spindle shaped cell morphology.

**Figure 6:**
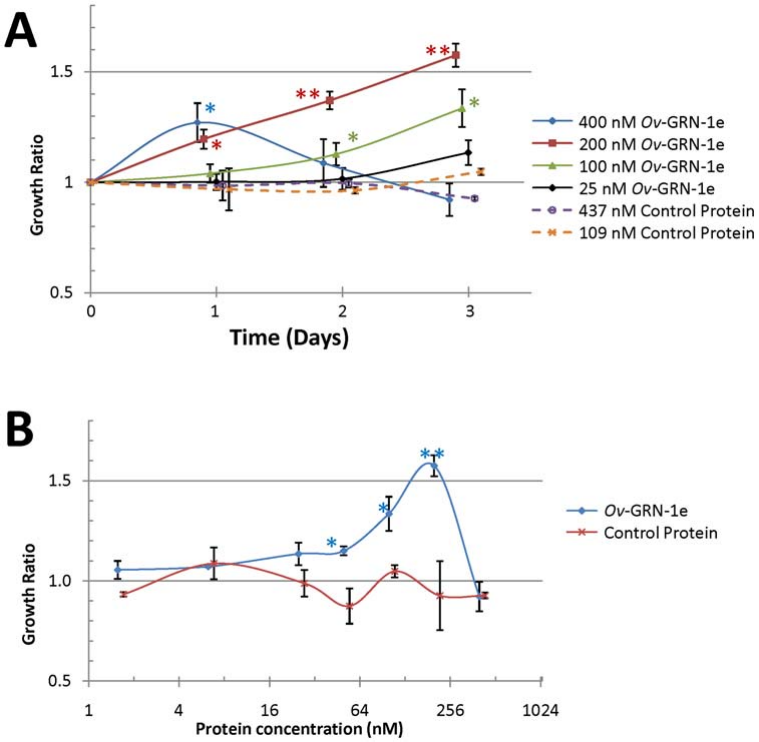
Recombinant refolded *Ov*-GRN-1e stimulates growth of NIH-3T3 fibroblasts at nanomolar concentrations as measured by WST-1 cell count assay. Growth is expressed as a ratio of cell numbers after incubation with recombinant proteins compared to PBS (growth rate = 1.0). Panel A: The effects of *Ov*-GRN-1e and control protein on NIH 3T3 cells for 3 days. To facilitate view of error bars, data points were offset by 0.05 units. Panel B: Growth of cells after three days of culture in the presence of 2–400 nM *Ov*-GRN-1e and *Sm*-TSP-2 control protein. All curves are the averages of duplicates and standard deviations are shown as error bars at each data point. Asterisks denote significance at the *P*<0.05 (*), *P*<0.01 (**) and *P*<0.001 (***) levels between growth ratios of cells cultured with *Ov*-GRN-1e and negative control protein at equivalent concentrations.

A range of concentrations of recombinant *Ov*-GRN-1e was included with cells under different culturing conditions, based on information from other investigations with ES, as described above. Nanomolar concentrations (50–200 nM) of *Ov*-GRN-1e induced significant growth of cells above growth of control cells treated with either PBS or an irrelevant recombinant protein purified under the same conditions as for *Ov*-GRN-1e (Figures 6 and S4). Four hundred nM *Ov*-GRN-1e induced significant growth after one day (*P*<0.05), after which cell growth slowed and cell numbers were equivalent to cells treated with control protein by day 3 (Figure 6A). At higher concentrations (≥800 nM) the cells suffered adverse effects and did not survive beyond 24 hr (not shown). Compared to control protein, 200 nM *Ov*-GRN-1e promoted significant growth after one day (*P*<0.05) and 50–200 nM *Ov*-GRN-1e caused significant growth by day 3 (*P*<0.05; Figure 6B). Cell proliferation induced by both ES products and recombinant *Ov*-GRN-1 was completely ablated in the presence of 10 µM U0126, an inhibitor of Erk1/2 signalling, indicating that *Ov*-GRN-1, like human PGRN, signals via the MAPK pathway.

**Figure 7:**
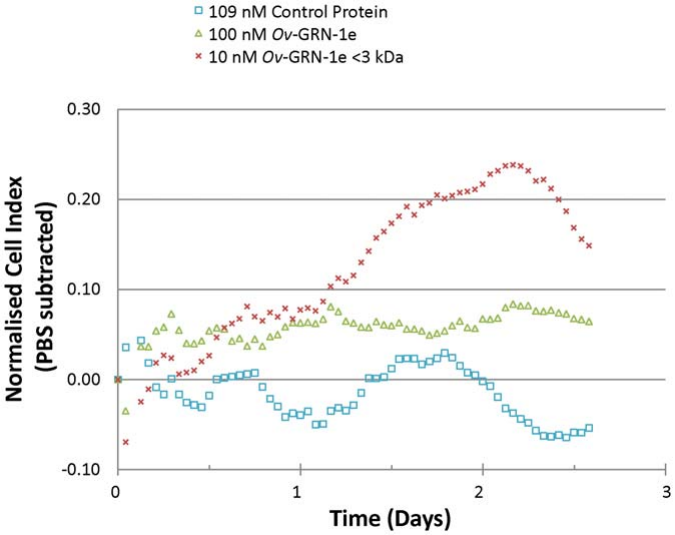
Refolded recombinant *Ov*-GRN-1e stimulates growth of NIH-3T3 fibroblasts at nanomolar concentrations as measured in real time using an xCELLigence system (Roche). The curves represent the difference between control cells grown in medium plus PBS versus test cells (recombinant proteins added) as measured by Cell Index; data have been normalized prior to sample addition. The control protein and *Ov*-GRN-1e were added to cells at equimolar quantities, as previously determined and optimized using the WST-1 assay. *Ov*-GRN-1e before and after (column flow-through) filtration through a 3 kDa cut-off nanosep filter are shown.

Intriguingly, refolded *Ov*-GRN-1e that was concentrated (retentate) using a 3 kDa cut-off centrifugal concentrator membrane did not induce cell growth, however the column flow through (i.e. >3 kDa) did induce proliferation. Indeed, the proliferation was greatly enhanced at lower concentrations (10 nM) compared with refolded *Ov*-GRN-1e that had not undergone concentration (50–200 nM; measured in real time by xCELLigence - Figures 7 and S5). Using both SDS PAGE and Western blotting with anti-6×His antibody we identified a small amount (∼5–10% of purified and refolded protein) of refolded protein that reproducibly passed through a 3 kDa cut-off membrane.

### Anti-*Ov*-GRN-1 antibodies block cell proliferation induced by *O. viverrini* ES products

**Figure 8:**
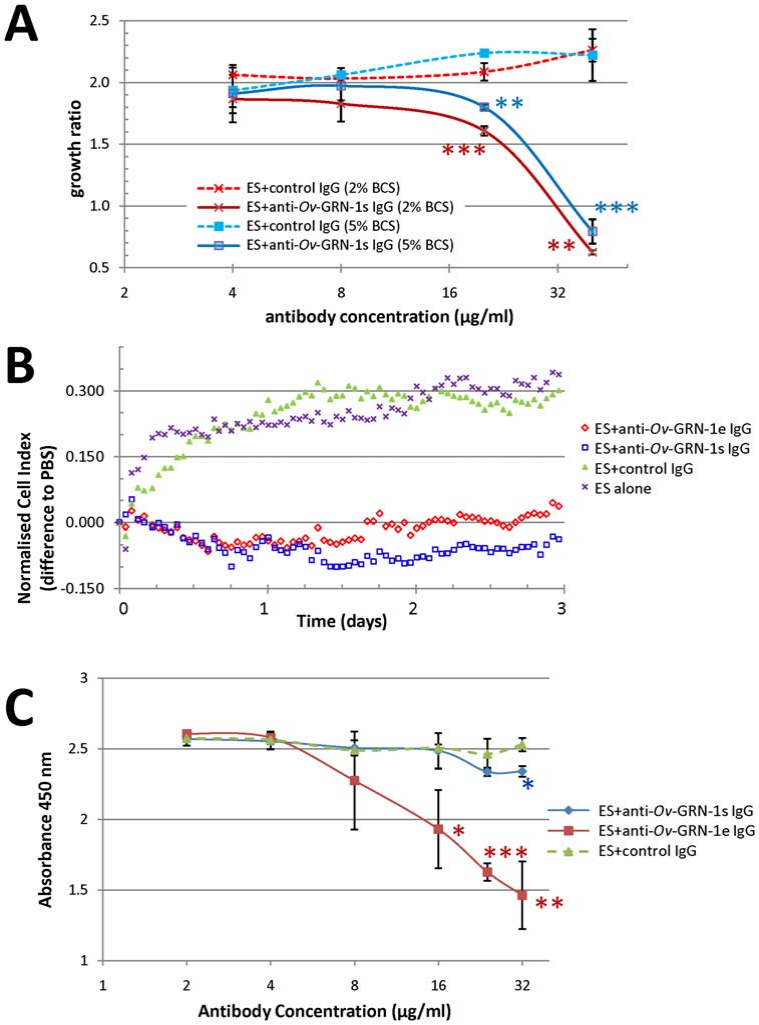
Inhibition of ES-induced cell proliferation by anti-*Ov*-GRN-1 IgG. Panel A: Anti-*Ov*-GRN-1s IgG inhibited growth in a dose-dependent manner of NIH-3T3 fibroblasts induced by *O. viverrini* ES products as measured by the WST-1 metabolic assay. Panel B: Anti-*Ov*-GRN-1e and anti-*Ov*-GRN-1s IgGs inhibited growth of NIH-3T3 fibroblasts induced by *O. viverrini* ES as measured in real time using an xCELLigence system (Roche). Panel C: Anti-*Ov*-GRN-1e and anti-*Ov*-GRN-1s IgGs inhibited growth of KKU-100 cholangiocarcinoma cells induced by *O. viverrini* ES in a dose-dependent manner. Data were obtained using the WST-1 metabolic assay. All data shown are means of triplicate experiments and standard deviations at each data point are shown as error bars. Asterisks denote significance at the *P*<0.05 (*), *P*<0.01 (**) and *P*<0.001 (***) levels between growth ratios or absorbance readings of cells cultured with anti-*Ov*-GRN-1 and negative control antibodies at equivalent bovine calf serum concentrations.

To determine whether *Ov*-GRN-1 was responsible for the mitogenic activity of ES, we attempted to neutralize the mitogenic activity of ES with anti-*Ov*-GRN-1 antibodies. Anti-GRN-1s IgG inhibited ES-induced proliferation of NIH-3T3 fibroblasts (Figures 8A and S6A). After 3 days of cell culture, significant inhibition of proliferation was evident at concentrations of 20 and 40 µg/ml IgG in both 2% and 5% BCS cultures (*P*<0.01 - <0.001). Similar antibody-induced suppression of proliferation was obtained in the absence of BCS over two days (not shown), but after two days control cells (treated with PBS) began to die.

NIH-3T3 cells were grown under identical conditions as described above −20 µg/ml ES, 20 µg/ml test or control IgGs and 2% BCS over 3 days - and monitored with the xCELLigence system (Figures 8B and S6B). Cells treated with ES alone or ES in the presence of control IgG grew at the same rate (F_(2,186)_ *P* = 0.65) with a steady increase of 0.2–0.25 units over 24 hours and then a reduced rate of increase of 0.05–0.1 units for the subsequent two days. This was similar to the growth rates measured with WST-1. When ES in the presence of either anti-*Ov*-GRN-1e or anti-*Ov*-GRN-1s IgGs were incubated with cells, growth slowed significantly (F_(2,186)_ *P*<0.0001) compared to cells treated with ES alone or ES plus control IgG and showed only minor variations from the growth profile of NIH-3T3 cells treated with PBS alone (Figure 8B).

The inhibitory effect of anti-*Ov*-GRN-1 IgG on the growth of the KKU100 CCA cell line induced by ES products was also assessed. Cells were cultured for 3 days in RPMI 1640/2% BCS with a final concentration of 20 µg/ml ES. Cell growth is presented as absorbance at 450 nm rather than as a growth ratio because of the differences in growth characteristics between this cell line and NIH-3T3 fibroblasts. The same general trend was observed, whereby anti-GRN-1e and to a lesser but still significant extent, anti-GRN-1s, IgGs inhibited proliferation induced by ES in an antibody dose-dependent fashion (Figure 8C). Significant inhibition was observed with 16 µg/ml (*P*<0.05) and 24 µg/ml (*P*<0.001) anti-GRN-1e IgG. Addition of IgGs to cells in the absence of ES had no effect on cell growth (not shown).

## Discussion

Initiation of CCA in chronic opisthorchiasis in humans [28] and experimentally infected hamsters [29] is thought to be multi-factorial, involving (1) infection-induced inflammation, particularly the release of reactive oxygen and nitrogen species from inflammatory cells, (2) dietary nitrosamines consumed by endemic populations, (3) secretion by the fluke of mitogens into the biliary tree [5]. Co-culture of *O. viverrini* adult worms with mouse fibroblasts (NIH-3T3) where parasites and cells are separated by a porous membrane results in cell proliferation [4]. Here we show that soluble ES products, in the absence of live *O. viverrini* parasites, and recombinant *Ov*-GRN-1e cause proliferation of mouse fibroblasts and a human CCA cell line, and that proliferation caused by ES products can be blocked with anti- *Ov*-GRN-1e antibodies. This data implies that *Ov*-GRN-1 is perhaps the major mitogenic factor in ES, and this protein contributes to the development of an environment that is conducive to CCA.

The GRN protein family is found in a diverse range of organisms including bacteria, plants and animals. Our phylogenetic analysis of the protein family suggested that a majority of GRN proteins do not form clades based on taxonomic groupings but rather group according to protein functions. The individual GRN domains from human PGRN form distinct clades with homologues from other species, supporting the notion that these proteins have evolved to perform distinct functions in different organisms, and furthermore, individual GRN domains released after processing of the multi-domain PGRN have also evolved to perform discrete functions. A range of organisational archetypes are seen within the family, ranging from single GRN domains behind a secretory signal peptide, as seen in earthworms and the liver flukes, to multi-homodomain PGRNs and even single GRN domains fused to other protein domains [7].

The structure of GRN is unique, although it can be partially superimposed on the 3-dimensional fold of epidermal growth factor (EGF), despite the absence of primary sequence identity [30]. Furthermore, Ov-GRN-1 and human PGRN, like EGF, triggers similar signalling cascades, including the MAPK pathway [31]. When NIH-3T3 fibroblasts were co-cultured with *O. viverrini* adult worms without serum, only mRNAs associated with EGF and TGF-beta signalling pathways were significantly upregulated, further supporting a role for *Ov*-GRN-1 in parasite-induced proliferation and downstream signalling of host cells [32]. Granulin-induced cell proliferation can result in upregulation of EGF family members, such as VEGF, which could account for upregulation of genes involved in the EGF pathway [31].

*Ov*-GRN-1 was expressed at very low levels in lepidopteran (*Sf*9) cells, limiting more thorough investigation of this form of the protein. By contrast, *Ov*-GRN-1 was expressed at high yield in *E. coli* and could be refolded into an active form that induced proliferation at nanomolar concentrations. This is the first report, to our knowledge, of a secreted growth factor from a parasite that induces proliferation of host cells. This is also the first report of functional recombinant expression of a single domain granulin. Tolkatchev expressed all 7 granulin domains individually from human PGRN but only three, granulins A, C and F, appeared to adopt at least partially correct fold and induced growth [33]. Despite refolding denatured *Ov*-GRN-1e to generate a functional recombinant protein that induced cell proliferation, the majority of functional recombinant protein passed through a 3 kDa cut-off membrane. If *Ov*-GRN-1 does indeed adopt a similar fold to carp granulin (Figure 2C), the super helical structure held tightly together by 6 disulphide bonds might well pass through a 3 kDa membrane (Figure 7). Furthermore, the apparent molecular weights of native human granulins purified from leukocytes range from 1.7–3.2 kDa and would likely pass through a 3 kDa membrane, as we observed here with functionally active *Ov*-GRN-1 [34]. The low nanomolar activity displayed by refolded *Ov*-GRN-1 is comparable to the activity of purified human granulins [15].

GRN is associated with many aggressive cancers. It is over-expressed in human liver [14],[19], renal [35], breast [31],[36],[37], bladder [16] and brain [15] tumors. It may promote cancer progression by stimulating angiogenesis, suppressing anoikis (a form of apoptosis), promotion of tumor invasion and anchorage independence, all of which support tumor expansion in the unfavourable interstitial environment [7],[16],[17]. Preventing the activity of PGRN in a range of tumor types, either through gene silencing or antibody neutralization, reduces or entirely inhibits tumor progression [18]. Transfection of fibroblasts with PGRN induces serum independent proliferation but does not transform them into neoplastic cells, suggesting that the protein is probably not oncogenic by itself, but over-expression of PGRN in the SW-13 non-malignant adrenal carcinoma cell line made it highly tumorigenic [38]. Indeed, PGRN is a therapeutic target for liver cancer, particularly HCC. An anti-PGRN monoclonal antibody inhibited tumor growth *in vivo* in nude mice transplanted with human HCC [19]. The anti-PGRN antibody also inhibited growth of hepatoma cells but had no significant effect on normal liver cells, and inhibited the growth and proliferation of established tumors via the p44/42 MAPK and Akt pathways. These findings demonstrate that GRN is an important factor in the initiation of liver cancer and the migration of cancerous cells. We showed here that *Ov*-GRN-1 signals via the MAPK pathway, further accentuating the potential role of this parasite protein in the initiation of CCA in people with chronic opisthorchiasis.

Increased PGRN expression has not been reported in CCA, however, when gene expression profiles from intrahepatic CCA associated with or without *O. viverrini* were compared, genes associated with growth factor signalling were the most highly upregulated ontology in the non-fluke associated CCA, whereas genes involved in xenobiotic metabolism were the most highly upregulated genes in fluke associated CCA [39]. It is intriguing that genes involved in growth factor signalling pathways were selectively upregulated in non-fluke associated CCA. This prompts the speculation that *Ov*-GRN-1 causes excessive proliferation and migration of pre-cancerous and cancerous cells in the bile ducts of infected people, obviating the necessity for local upregulation of the host growth factors and associated signalling molecules during tumorigenesis.

Why *O. viverrini* secretes such a potent growth factor that acts on host cells is unclear. One potential role for fluke GRN is in the wound repair. Inflammatory cells secrete peptides derived from PGRN [34], and PGRN mRNA is highly induced in dermal fibroblasts and epithelial cells following transcutaneous puncture wounds [8]. Furthermore, recombinant PGRN increased the accumulation of inflammatory cells, blood vessels and fibroblasts at puncture sites, implying a direct role as a wound-healing growth factor [9]. *O. viverrini* adult worms grasp the bile duct wall with their suckers and feed on the biliary cells, often severely damaging the epithelium. Additional inflammation occurs as a result of the local immune response to resident worms (reviewed in [5]). *Ov*-GRN-1 might therefore play a role in wound repair at and around the feeding site to minimize the pathology that the parasite causes to the host. Another potential role for *Ov*-GRN-1 is in the “farming” of host cells for nutritional purposes. By promoting growth of cells at the feeding site, the parasite is ensured of a steady supply of nutrients. Blood-feeding leeches secrete a GRN that inhibits thrombin activity [40], and *Ov*-GRN-1 might also perform a similar function to interfere with clot formation while feeding.

Like some other *O. viverrini* proteins, *Ov*-GRN-1 was identified on the surface of and inside host biliary epithelial cells. *O. viverrini* ES products adhere to and are internalised by hamster biliary epithelial cells in the first order bile ducts as well as the small extra-hepatic bile ducts where the parasite is too large to reside [41]. Until now, only one ES product that is internalized by host cells had been identified - thioredoxin peroxidase [42]. The mechanism of uptake of *O. viverrini* ES components by host cells is unknown. With a related fluke, *Schistosoma japonicum*, fluke glutathione transferase (GST) is translocated from the medium into a variety of mammalian cell types via an endocytotic pathway involving clathrin-coated pits [43]. Like most helminth parasites, *O. viverrini* secretes a GST (J. Mulvenna et al., unpublished), which along with other ES proteins (such as *Ov*-GRN-1), might enter host cells via a similar endocytotic mechanism. Translocation of *O. viverrini* proteins into host biliary epithelial cells is particularly important due to the carcinogenic nature of this parasite and the putative roles that internalised ES play in transforming host cells [5]. *Helicobacter pylori* delivers the CagA protein into gastric epithelial cells where it interacts with a kinase involved in cell polarity, resulting in disorganization of gastric epithelial architecture, inflammation and carcinogenesis [44]. Translocation of *O. viverrini* ES products such as *Ov*-GRN-1, into biliary epithelial cells might likewise interfere with signalling, promoting carcinogenesis. Moreover, liver fluke ES products inhibit apoptosis (B. Sripa, unpublished; [45]), further contributing to a tumorigenic environment.

Despite the deployment of mass drug administration programs throughout Thailand, opisthorchiasis is still a major public health concern, and the prevalence of the infection in some areas is increasing [5],[46]. Like other neglected tropical diseases, an integrated control program is required to have a lasting impact on reducing transmission and disease burden. To this end, a vaccine for opisthorchiasis is desperately needed to reduce worm burdens and minimize pathology. A recombinant vaccine based on *Ov*-GRN-1 is particularly attractive because of the potential role of this protein in establishing a pro-tumorigenic environment in the bile ducts. Such a vaccine would therefore have a major impact on reducing both parasite burdens and the incidence of CCA, the most prevalent and fatal of the liver cancers in north-east Thailand.

## Materials and Methods

### Ethics statement

Hamsters used in this study were maintained at the animal research facility of the Khon Kaen University Faculty of Medicine; all work was conducted in accordance with protocols approved by the Khon Kaen University Animal Ethics Committee. Mice used in this study were housed at the Queensland Institute of Medical Research (QIMR) animal facility; all work was conducted in accordance with protocols approved by the QIMR Animal Ethics Committee.

### Preparation of parasite extracts

*O. viverrini* metacercariae were obtained from naturally infected cyprinoid fish in Khon Kaen province, Thailand. The fish were digested with pepsin-HCl, washed and used to infect hamsters (*Mesocricetus auratus*) by stomach intubation. Adult *O. viverrini* worms were recovered from bile ducts of euthanized hamsters infected for 3 months. Somatic adult worm extract (SAE) was prepared from frozen and homogenized adult worms resuspended in PBS with a cocktail of protease inhibitors covering serine, aspartic, cysteine and metallo-proteases (protease inhibitor cocktail set #5, Roche). ES products were prepared from live adult worms washed in antibiotics and incubated in modified RPMI-1640 (Invitrogen) at 37°C/5% CO_2_. Supernatant containing the ES products was harvested daily for 7 days [41]. The supernatant was concentrated 20-fold to 100–300 µg/ml with 3 kDa Jumbosep spin concentrators (Pall) and aliquoted for storage at −80°C.

### Identification of *Ov*-GRN-1 in *O. viverrini* ES products using LC-MS-MS

*Ov*-GRN-1 was identified in the ES products of adult *O. viverrini* using liquid chromatography tandem mass spectrometry (LC-MS/MS). The proteins present in ES products were identified by trypsin digestion followed by a combination of off-gel electrophoresis and LC-MS/MS as described by us for the analysis of ES products from the hookworm, *Ancylostoma caninum* [47]. For multiple reaction monitoring (MRM), recombinant *Ov*-GRN-1 in water and *O. viverrini* ES were digested as described [47]. A Dionex 3000 HPLC system (Dionex) was used to perform reversed phase separation of the samples using a C18 300A column (150 mm×2 mm) with a particle size of 5 µm (Phenomenex). Twenty microliter aliquots of samples were dissolved in 5% formic acid (aq) and injected onto the HPLC column. The mobile phase consisted of solvent A (0.1% formic acid (aq)) and solvent B (90/10 acetonitrile/0.1% formic acid (aq)). Tryptic peptides were eluted using a gradient elution programme of 0–40% B in 40 min, 40–80% B in 10 min and finally a 5-min hold at 80% B, followed by a return to 0% B for a 10-min equilibration. The flow rate was 250 µl/min. Eluate from the RP-HPLC column was directly introduced into the TurboIonSpray source. Mass spectrometry experiments were performed on a hybrid quadrupole/linear ion trap 4000 QTRAP MS/MS system (Applied Biosystems). All analyses were performed using MRM, Information Dependant Acquisition Initiation Enhanced Product Ion experiments using both the triple quadrupole and the linear ion trap acquisition modes. Analyst 1.5.1 software was used for data analysis. The acquisition protocol to provide mass spectral data for both identification and characterization involved monitoring the HPLC eluant using MRM scans; ions over the background threshold of 200 counts per second were subjected to examination using the enhanced resolution scan to confirm charge states of the multiply charged molecular ions. The most and next most abundant ions in each of these scans with a charge state of +2 to +4 or with unknown charge were subjected to collision induced disassociation using a rolling collision energy dependent upon the m/z and the charge state of the ion. An enhanced product ion scan was then used to acquire the product ion spectrum. The 4000 QTRAP equipped with a TurboIonSpray Source was operated in the positive electrospray ionization mode.

### Sequence analysis

Sequences were edited and analysed with assistance from the MacVector software package. Homology searches were performed using Blast search at NCBI (http://blast.ncbi.nlm.nih.gov/Blast.cgi). ORFs were analysed for signal peptides/anchors using SignalP-NN prediction and SignalP-HMM prediction at http://www.cbs.dtu.dk/services/SignalP/. The *O. viverrini* cDNA encoding for GRN was termed *Ov-grn-1* and was submitted to GenBank under accession number FJ436341. The structural architecture of GRN family members was obtained from entry IPR000118 at the Interpro 18.0 database [48]. Prediction of potential glycosylation sites was determined using the YingOYang server [49].

### Structural modelling

Using version 3 of MODELLER, the three-dimensional structure of *Ov*-GRN-1 was predicted based on comparative modelling to carp granulin [23]. The covalent geometry of the modelled structure was in agreement with the template structures with all but one of the residues occupying the allowed regions of the Ramachandran plot. The quality of the stereo-chemical structures of the models was determined using PRO-CHECK-NMR [24]. Pymol was used to view the homology models (http://www.pymol.org)

### Phylogenetic tree

The phylogenetic relationship of *Ov*-GRN-1 with other GRN family members was inferred using a neighbor joining analysis in PAUP beta version 8.0 for Macintosh. Bootstrap values were determined from 1000 replicates. Where bootstrap values were below 50%, clades were collapsed to form polytomies. Where multiple GRN domains were observed within one PGRN protein (e.g., vertebrates and schistosomes), individual GRN domains sharing the greatest identity with *Ov*-GRN-1 were selected and numbered 1–3 in order of their identities. The individual GRN domains of human PGRN, however, have been alphabetically designated A–G in the order GFABCDE, based on their description in the literature.

### RNA extraction and RT-PCR

Extraction of *O. viverrini* RNA and subsequent reverse transcription PCR (RT-PCR) was carried out as described [50], with minor modifications. Total RNA from each developmental stage of *O. viverrini* was extracted with Trizol (Invitrogen) according to the manufacturer's instructions. Contaminating genomic DNA was removed by treatment of RNA with DNase I (Promega). For RT-PCR, first-strand cDNA was synthesized from 1.0 ug of total RNA using avian myeloblastosis virus reverse transcriptase (Promega) and an oligo (dT) primer at 42°C for 60 min. A 1.0 µl aliquot of the cDNA was amplified using primers specific for the control beta-actin mRNA (Forward CGAGGTATCCTCACCCTCAA, Reverse GCGACTCGCAACTCATTGTA) and the target *Ov-grn-1* mRNA (Forward CGCGCGCCATGGATACTTTGCAGCCAATT, Reverse GCGCGCCTCGAGTGCGACCTTTCGAGCGTT) based on the following conditions: 30 sec denaturation at 94°C, 30 sec annealing at 55°C, and 30 sec extension at 72°C for 30 cycles. Control RT-PCR reactions were performed without reverse transcriptase to ensure that amplified products were derived from cDNA and not contaminating genomic DNA. PCR products were sized by electrophoresis through 1% agarose and visualized under UV light after staining with ethidium bromide.

### Recombinant protein expression

*Ov*-GRN-1 was expressed in both bacterial and insect cell expression systems. The complete ORF minus the predicted signal sequence was amplified using Expand polymerase (Roche) from an adult *O. viverrini* cDNA library [6] using primers described below and cloned into the plasmid pMIB/V5-His (Invitrogen) for insect (*Spodoptera frugiperda*) cell expression or cloned into the *Nde*I and *Xho*I sites of the pET41a vector (Novagen) for *E. coli* expression, thereby removing the GST fusion tag but retaining the 6×His tag and allowing for native N-terminal protein expression. Primers for insect cell expression were: pMIB F3 HindIII CGCGCGAAGCTTAATGGATACTTTGCAGCCAATT; pMIB R3 XbaI GCGCGCTCTAGATGCGACCTTTCGAGCGTT. Plasmid preparation, cell transfection, colony selection and growth of *Sf*9 cells (Invitrogen) was as previously described [51]. Primers for *E. coli* expression were: pet41 NcoI F7 CGCGCGCCATGGATACTTTGCAGCCAATT; pet41 XhoI R7 GCGCGCCTCGAGTGCGACCTTTCGAGCGTT. Plasmids were prepared using standard techniques and used to transform *E. coli* cell lines (BL21, rosetta, rosetta-gami cells – Novagen) followed by selection with kanamycin and other appropriate antibiotics according to the manufacturer's instructions. Cells were grown in LB medium at temperatures between 16–37°C in 1 L Schott bottles at 220 rpm. Cultures were induced with 1 mM IPTG upon reaching an OD_600_ of 0.5 and grown overnight before harvesting the cell pellet.

### Purification of recombinant proteins

Since both recombinant proteins (derived from *E. coli* and *Sf*9 insect cells) contained C-terminal 6×His tags, the purification procedures included immobilized metal ion affinity chromatography (IMAC). Purification was undertaken using an AKTA basic purification system (GE) at 4°C. Henceforth we refer to the *E. coli*-derived recombinant protein as *Ov*-GRN-1e and *Sf*9-derived protein as *Ov*-GRN-1s. For *Ov*-GRN-1s, 3 L of culture supernatant was passed across a 5 ml Hitrap HS HP cation exchange column (GE) with a gradient of 0–1 M NaCl over 10 column volumes. Fractions enriched for recombinant protein were detected using an anti-V5 affinity tag antibody (Invitrogen). Further purification of these fractions was achieved by a subsequent IMAC step; protein was bound to 1 ml Ni-NTA resin (Qiagen) in 10 mM imidazole/sodium phosphate (pH 8) and washed with 10 column volumes of 20, 40 and 60 mM imidazole, followed by elution in 250 mM imidazole. Fractions containing recombinant protein were subjected to a second round of IMAC as above, followed by concentration to 1 mg/ml and buffer exchange into PBS using 3 kDa microsep spin columns (Pall). For purification of *Ov*-GRN-1e, 3 g of *E. coli* cell pellet was resuspended in 30 ml binding buffer (50 mM Tris-HCl, 300 mM NaCl, 0.1% Triton X-100) followed by three disruption cycles through a chilled French press at 16–18000 psi. The sample was centrifuged at 4000 *g* and the pellet was dissolved in 6 M urea in nickel (Ni)-NTA binding buffer with 40 mM imidazole overnight at 4°C with gentle mixing. The supernatant was collected by centrifugation as above and purified by denaturing IMAC, with 6 M urea in all buffers, over a 5 ml His-trap Ni-IDA column (GE). The column was washed with 100 mM imidazole and recombinant protein eluted with 500 mM imidazole/6 M urea. The eluate was concentrated to 20 mg/ml using 3 kDa 15 ml Amicon Ultra centrifuge concentration devices (Millipore) and refolded by drop wise addition to a 20-fold greater volume of a range of refolding buffers [52]. The soluble material was buffer-exchanged into PBS using a PD10 column (GE).

### Antibody production and purification of IgG

*Ov*-GRN-1e and GRN-1s were adjusted to 0.4 mg/ml and diluted 1∶1 with Freund's adjuvant and emulsified. Twenty micrograms of protein (100 µl of protein∶adjuvant) was injected subcutaneously into each of four BALB/c female mice every two weeks, using Freund's complete adjuvant for the first immunization and Freund's incomplete adjuvant for the second and third immunizations. Two weeks after the final immunization, mice were euthanized, blood collected by cardiac puncture, and sera recovered from the clotted blood. Control sera were obtained from mice that were either (1) unimmunized or (2) immunized with an irrelevant control protein (recombinant *Na*-GST-1 glutathione-S-transferase from *Necator americanus*) [53]. Sera were pooled and diluted 1∶20 with PBS for affinity purification of IgG on Hitrap protein G (GE) at 4°C using an AKTA basic FPLC. Eluted fractions were concentrated with 30 kDa nanosep spin concentrators (Pall), buffer exchanged into PBS and stored at −80°C.

### Western blots

Denaturing and native polyacrylamide gel electrophoresis (PAGE) was performed using 15% gels. Proteins were electro-transferred to Biotrace nitrocellulose membranes (Pall), which were then sliced into 3 mm wide strips. Membranes were blocked in 5% skimmed milk powder (SMP)/PBS with 0.05% Tween 20 (PBST) for 60 min at room temperature (RT) with gentle shaking and probed overnight with 8 µg/ml mouse IgG diluted in 2% SMP/PBST at 4°C. Subsequently, membranes were washed (3×10 min each) in PBST at RT followed by probing with horse radish peroxidase (HRP)-conjugated goat anti-mouse IgG (Zymed Laboratories) diluted 1∶300 in 2% SMP/PBST at RT with gentle rocking for 60 min. After washing to remove the secondary antibody, colorimetric signals were developed in the presence of hydrogen peroxide and diaminobenzidine (DAB) (Pierce).

### Immunolocalization

*O. viverrini* adult worms or liver tissue from hamsters infected with *O. viverrini* (weeks 12–16 weeks) were fixed and cut by microtome into sections of 4 µm [41]. The sections were deparaffinised in xylene, hydrated in a series of ethanol and distilled water, respectively. Endogenous peroxidise was eliminated by incubating sectioned tissues in 5% H_2_O_2_ in methanol for 30 min, after which the sections were rehydrated in water and PBS. Non-specific staining was blocked by incubation in 5% normal mouse serum in PBS for 30 min. The sections were probed with pooled mouse purified IgG diluted 1∶100–1∶500 (v/v) in PBS and incubated overnight at 4°C. After rinsing 3×5 min with PBS the sections were incubated with horseradish peroxidase-conjugated goat anti-mouse IgG (Zymed Laboratories) for 1 h. Sections were rinsed with PBS 2×10 min, after which the slides were developed with DAB. The sections were counterstained with Mayer's haematoxylin, dehydrated, cleared in xylene and mounted in Permount® (Cen-Med). The sections were examined by light microscopy and images captured with a digital camera.

### Reagents for cell proliferation assays

ES products, recombinant proteins and IgG antibodies that were included in cell culture were filtered under sterile conditions with 0.22 µM syringe filters (Pall). Dilutions were carried out in sterile PBS in Twintec 96 well plates (Eppendorf). Samples were prepared so that 20 µl of ES protein or antibody in sterile PBS was added to 100 µl of cell culture media. Protein concentrations described hereafter refer to the final concentration in cell culture after dilution in media. All assays were conducted either in triplicate or duplicate, as specified in figure legends. Controls were relevant for the sample tested: expression matched recombinant proteins were included to assess effects of recombinant granulin – a hookworm protein, *Na*-ASP-2, expressed in insect cells [51] served as the control protein for *Ov*-GRN-1s; *Sm*-TSP-2 EC2 expressed in *E. coli* [54] served as the control protein for *Ov*-GRN-1e. Antibodies against recombinant proteins for use in cell culture were purified as above. The Erk1/2 (MAPK) kinase inhibitor, U0126 (Cell Signalling Technology) was used at a final concentration of 10 µM in some cell cultures to assess its ability to block signalling induced by ES products or recombinant *Ov*-GRN-1.

### Cell proliferation

NIH 3T3 mouse embryonic fibroblast cells (ATCC) were maintained as specified by ATCC protocols. Briefly, cells were maintained with regular splitting using 0.25% trypsin every 2–5 days in DMEM (Sigma) with 10% bovine calf serum (BCS) and 1×antibiotic/antimycotic (Invitrogen) at 37°C under 5% CO_2_. To assess the effects of ES on cell growth, multiple culture conditions were investigated in 96 well plates including seed densities (1,000–10,000 cells/well), cell attachment times before sample addition (3 h, 6 h, 16 h), BCS concentration (0, 2, 5 and 10%), ES concentration (10–80 µg/ml) and duration of culture after sample addition (1–3 days). The ability of live *O. viverrini* flukes to stimulate cell proliferation was assessed using a non-contact co-culture technique as described [4]. KKU-100 is a cell line derived from a human CCA and was maintained as described [55]. Cell proliferation was determined using two different approaches. First, cell numbers were assayed using the WST-1 cell proliferation reagent (Roche) as per product manual. Briefly the procedure involves incubating the cells with 10 ul of WST-1 for up to four hours at 37°C. During this period viable cells, which contain mitochondrial dehydrogenases, convert WST-1 to a water soluble formazan dye which has a peak absorbance at 450 nm which is measured on a Benchmark Plus plate reader (BioRad) and data were converted into cell numbers per well via comparison to standard curves.

Cell proliferation was also assessed by counting cell numbers in real time using a xCELLigence system and E plates (Roche) which monitors cellular events in real time by measuring electrical impedance across interdigitated gold micro-electrodes integrated on the bottom of tissue culture plates. The impedance measurement provides quantitative information about the biological status of the cells, including cell number, viability, and morphology [27]. Cell culture conditions tested were the same as those tested with the WST-1 dye (2% BCS, 20 µg/ml ES, 20 µg/ml IgG).

## Supporting Information

Figure S1 Reverse transcription PCR of different *Opisthorchis* life cycle stages. Lane 1: Egg (snail infective stage), Lane 2: Metacercariae (mammalian infective stage), Lane 3: Juvenile (two weeks maturation in liver), Lane 4: Young adult (one month), Lane 5: Adult (two months). Panel A shows *Ov-grn-1* RT-PCR and panel B shows actin control with equivalent lanes as in panel A. Negative controls without primers or without reverse transcriptase showed no bands (not shown).
(0.97 MB TIF)

Figure S2 Proliferation of host cells in response to *O. viverrini* live worms (A) and ES proteins (B). Data is shown as raw cell numbers and was used to generate growth ratios shown in Figure 5.
(0.56 MB TIF)

Figure S3 *O. viverrini* ES products induce growth of NIH-3T3 fibroblasts as measured in real time using an xCELLigence system. The curves represent the differences between each sample and the cells treated with PBS as measured by Cell Index (xCELLigence readout); data were normalized prior to sample addition.
(0.49 MB TIF)

Figure S4 Recombinant refolded *Ov*-GRN-1e stimulates growth of NIH-3T3 fibroblasts at nanomolar concentrations as measured by the WST-1 assay. Data is shown as raw cell numbers over three days in the presence of different concentrations of *Ov*-GRN-1 or control recombinant protein. Data was used to generate growth ratios shown in Figure 6.
(0.08 MB TIF)

Figure S5 Recombinant refolded *Ov*-GRN-1e stimulates growth of NIH-3T3 fibroblasts at nanomolar concentrations as measured in real time using an xCELLigence system (Roche). Data is shown as raw cell numbers and was used to generate growth ratios shown in Figure 7.
(0.51 MB TIF)

Figure S6 Inhibition of ES-induced proliferation of NIH-3T3 fibroblasts by anti-*Ov*-GRN-1 IgG as measured by the WST-1 assay (A) and in real time using an xCELLigence (B). Data is shown as raw cell numbers and was used to generate growth ratios shown in Figure 8.
(0.56 MB TIF)
