# Supplementary figures and images for: A Granulin-Like Growth Factor Secreted by the Carcinogenic Liver Fluke, Opisthorchis viverrini, Promotes Proliferation of Host Cells
Source: PLoS Pathog. 2009 Oct 9;5(10):e1000611. doi: 10.1371/journal.ppat.1000611 (PMC2749447; doi:10.1371/journal.ppat.1000611)

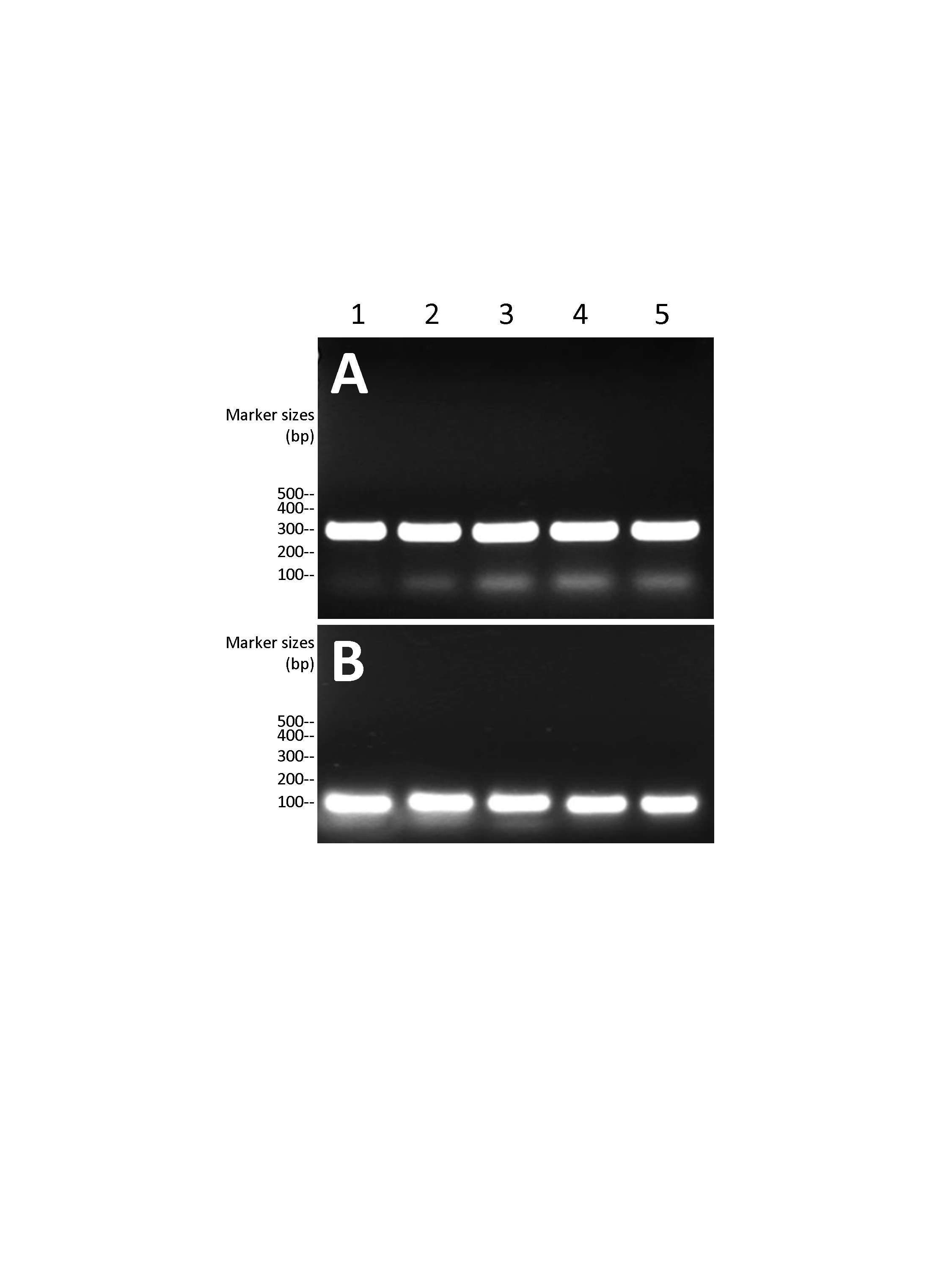

Supplement: Figure S1 — Reverse transcription PCR of different Opisthorchis life cycle stages. Lane 1: Egg (snail infective stage), Lane 2: Metacercariae (mammalian infective stage), Lane 3: Juvenile (two weeks maturation in liver), Lane 4: Young adult (one month), Lane 5: Adult (two months). Panel A shows Ov-grn-1 RT-PCR and panel B shows actin control with equivalent lanes as in panel A. Negative controls without primers or without reverse transcriptase showed no bands (not shown). (0.97 MB TIF) [file ppat.1000611.s001.tif]

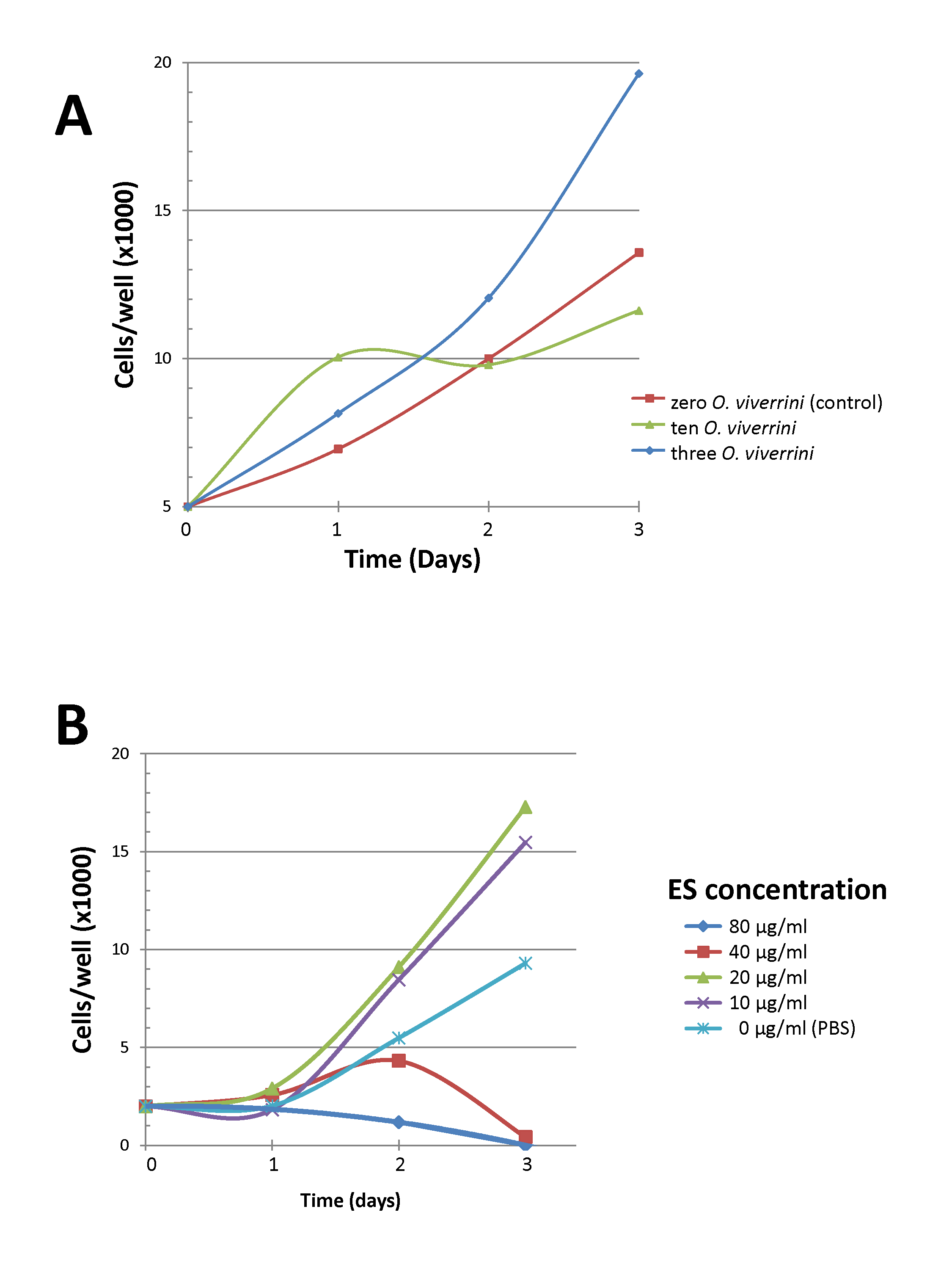

Supplement: Figure S2 — Proliferation of host cells in response to O. viverrini live worms (A) and ES proteins (B). Data is shown as raw cell numbers and was used to generate growth ratios shown in Figure 5. (0.56 MB TIF) [file ppat.1000611.s002.tif]

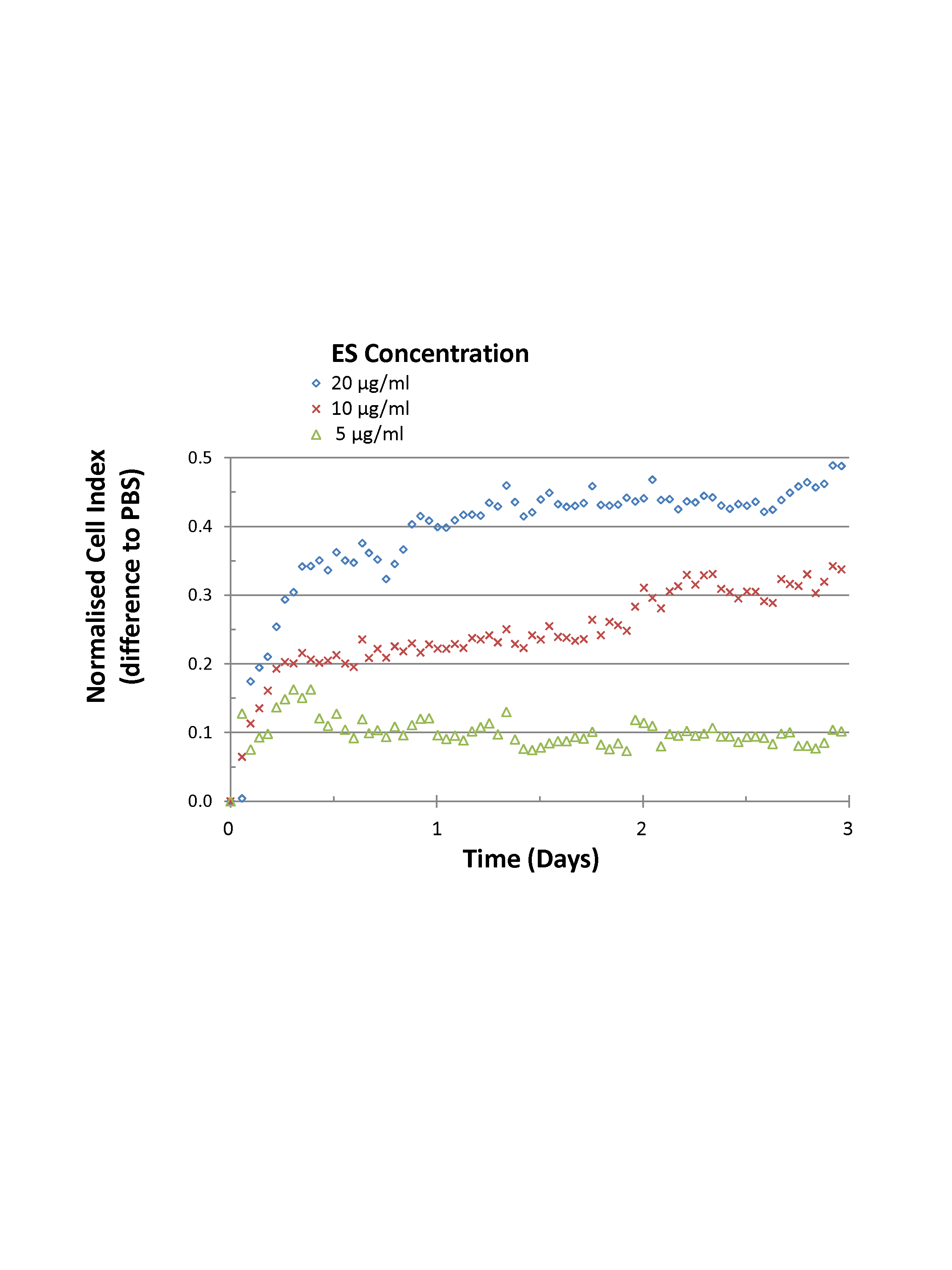

Supplement: Figure S3 — O. viverrini ES products induce growth of NIH-3T3 fibroblasts as measured in real time using an xCELLigence system. The curves represent the differences between each sample and the cells treated with PBS as measured by Cell Index (xCELLigence readout); data were normalized prior to sample addition. (0.49 MB TIF) [file ppat.1000611.s003.tif]

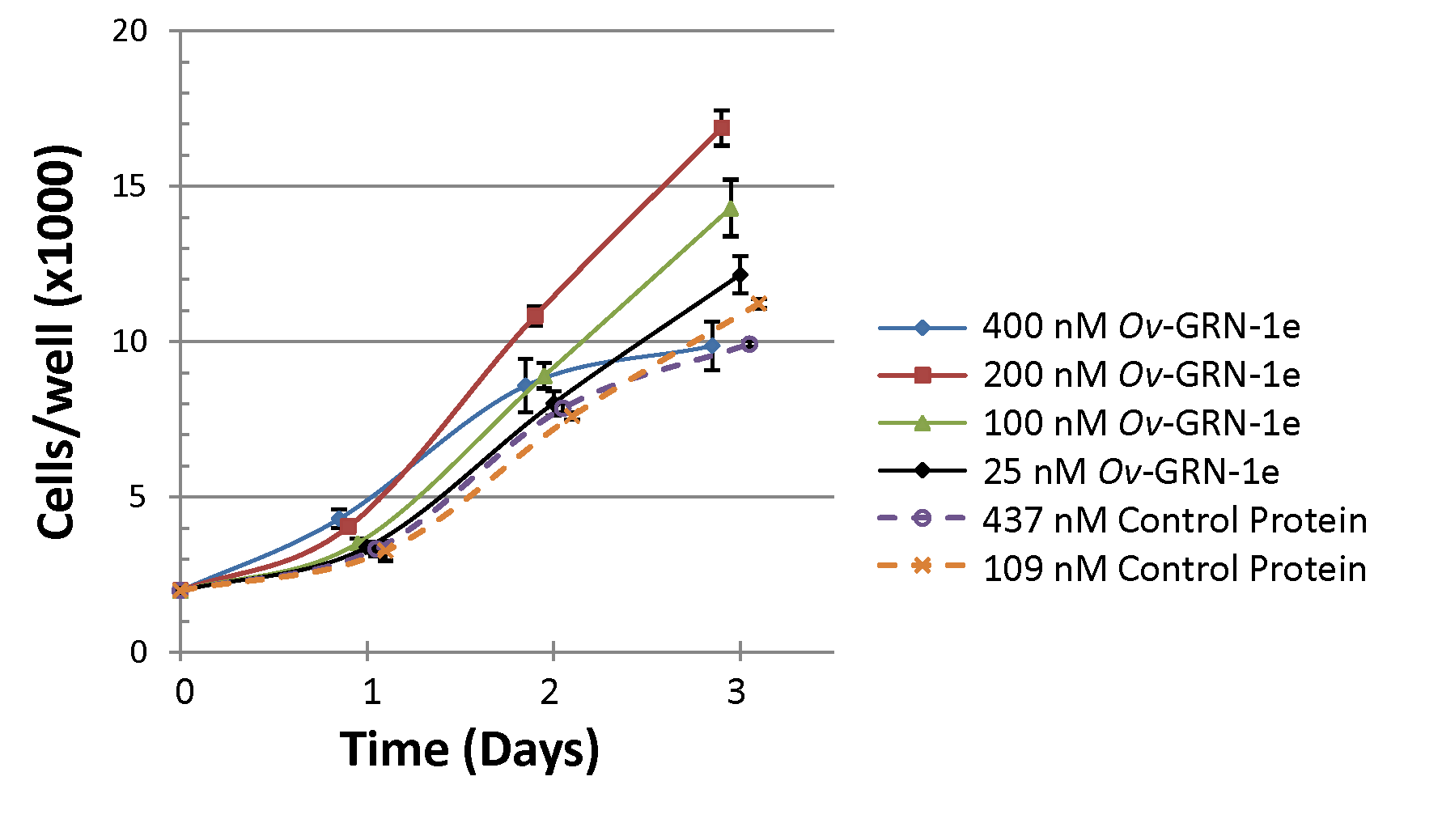

Supplement: Figure S4 — Recombinant refolded Ov-GRN-1e stimulates growth of NIH-3T3 fibroblasts at nanomolar concentrations as measured by the WST-1 assay. Data is shown as raw cell numbers over three days in the presence of different concentrations of Ov-GRN-1 or control recombinant protein. Data was used to generate growth ratios shown in Figure 6. (0.08 MB TIF) [file ppat.1000611.s004.tif]

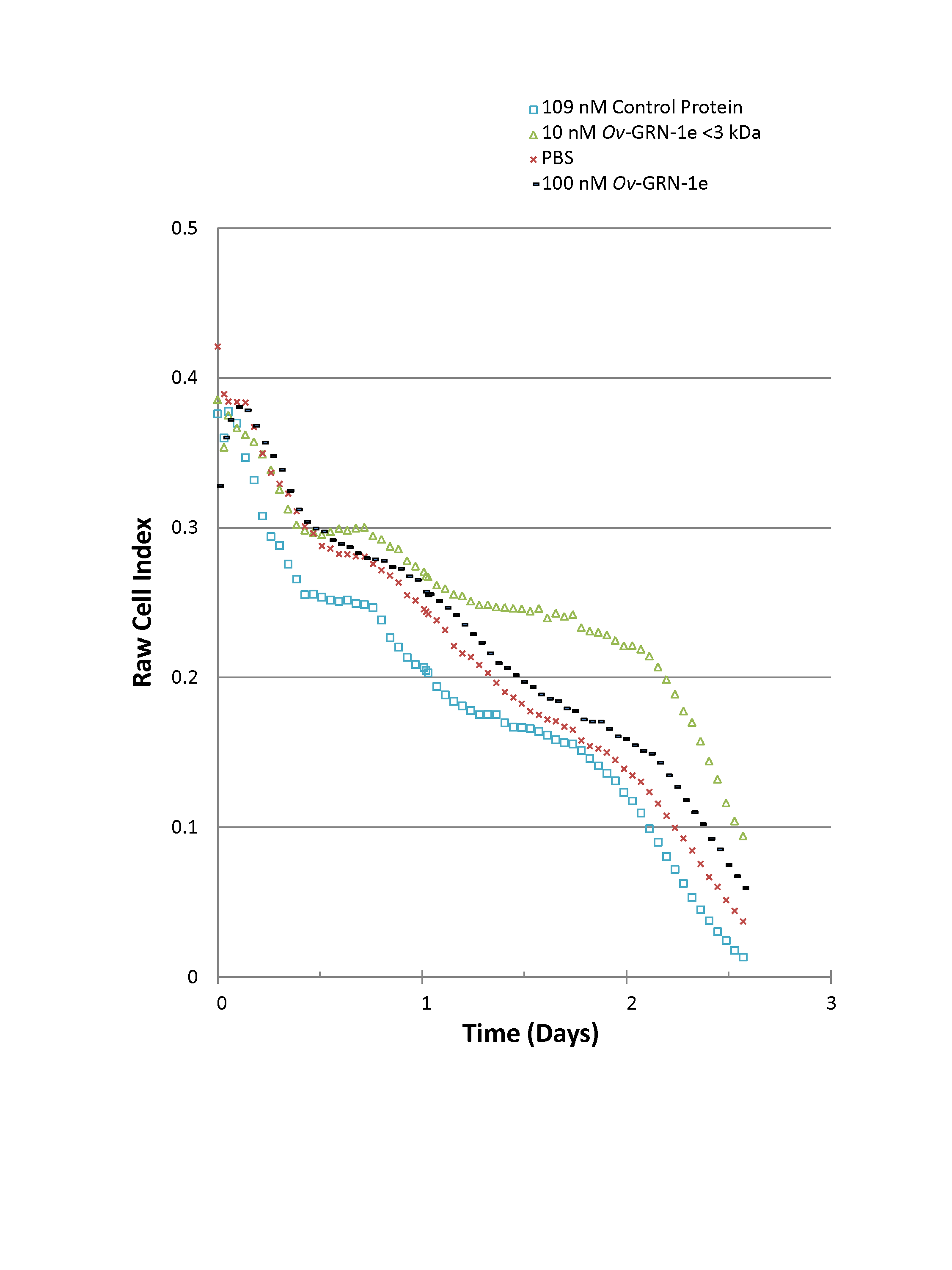

Supplement: Figure S5 — Recombinant refolded Ov-GRN-1e stimulates growth of NIH-3T3 fibroblasts at nanomolar concentrations as measured in real time using an xCELLigence system (Roche). Data is shown as raw cell numbers and was used to generate growth ratios shown in Figure 7. (0.51 MB TIF) [file ppat.1000611.s005.tif]

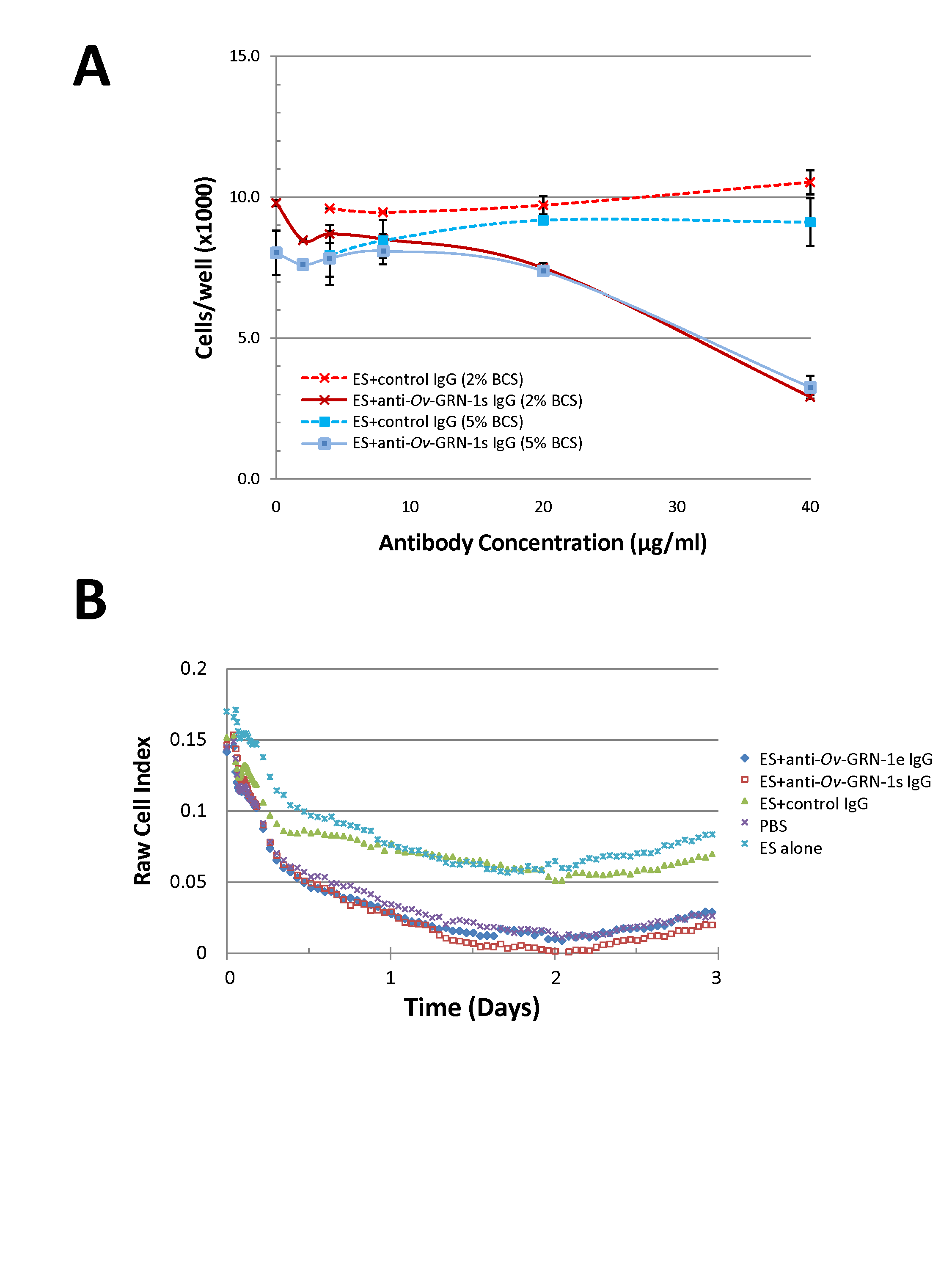

Supplement: Figure S6 — Inhibition of ES-induced proliferation of NIH-3T3 fibroblasts by anti-Ov-GRN-1 IgG as measured by the WST-1 assay (A) and in real time using an xCELLigence (B). Data is shown as raw cell numbers and was used to generate growth ratios shown in Figure 8. (0.56 MB TIF) [file ppat.1000611.s006.tif]
